# Biological and Clinical Effects of Calciprotein Particles on Chronic Kidney Disease-Mineral and Bone Disorder

**DOI:** 10.1155/2018/5282389

**Published:** 2018-03-27

**Authors:** Kenichi Akiyama, Takaaki Kimura, Kazuhiro Shiizaki

**Affiliations:** ^1^Division of Anti-Ageing Medicine, Center for Molecular Medicine, Jichi Medical University, Shimotsuke, Tochigi, Japan; ^2^Division of Renal Surgery and Transplantation, Department of Urology, Jichi Medical University, Shimotsuke, Tochigi, Japan; ^3^Division of Nephrology, Department of Internal Medicine, Jichi Medical University, Shimotsuke, Tochigi, Japan

## Abstract

Calciprotein particles (CPPs) are a new biological marker of chronic kidney disease-mineral and bone disorder (CKD-MBD). CPPs consist of phosphate, calcium, and some proteins, with phosphate being the major contributor to the level and biological activity of CPPs. Recent studies have shown the physiological and pathological significance of CPPs, including contributions to bone and mineral metabolism, and to tissue and organ impairments such as cardiovascular damage and inflammatory responses. These actions are well known as important aspects of CKD-MBD. Fibroblast growth factor 23 (FGF23), which is secreted from the bone as the phosphaturic hormone, is markedly elevated in CKD-MBD. Many clinical studies have shown significant relationships between the level of FGF23 and outcomes such as mortality, prevalence of cardiovascular disease, bone fracture, and levels of inflammatory markers. Basic and clinical studies have suggested that CPPs contribute to synthesis and secretion of FGF23. Surgical treatments such as renal transplantation and parathyroidectomy for patients with CKD-MBD suppress excess levels of phosphate, calcium, parathyroid hormone (PTH), and FGF23, which are related to the CPP level. Therefore, suppression of CPPs might also contribute to improved clinical outcomes after these treatments.

## 1. Introduction

**Figure 1:**
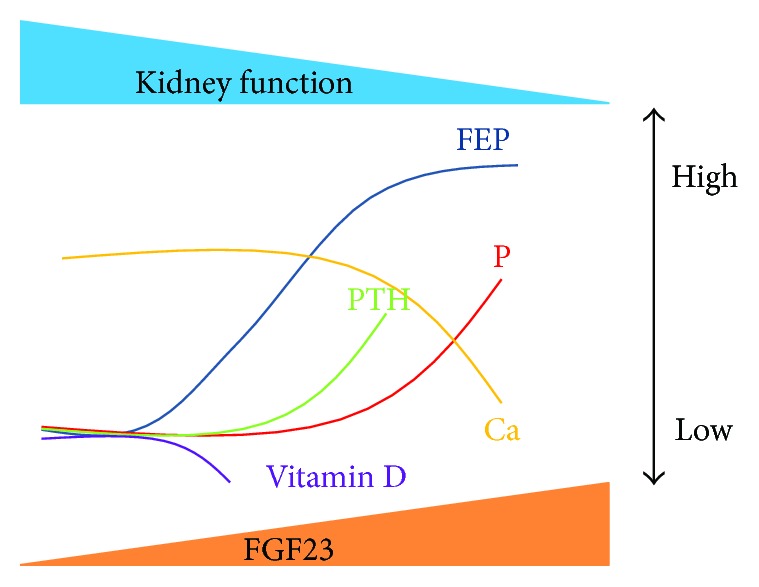
Changes of CKD-MBD-related markers accompanying decline of renal function. FEP and FGF23 levels show correlated elevation from the early to middle stages of CKD, with a subsequent decrease of 1,25(OH)_2_D_3_ and increase of PTH. When FEP cannot respond to the high level of FGF23 in end-stage CKD, the P and Ca levels start to elevate and decline, respectively.

Chronic kidney disease (CKD) has a high risk of complication with cardiovascular disorders, even before end-stage CKD requiring dialysis therapy. A major cause is soft tissue calcification due to impaired calcium (Ca) and phosphorus (P) metabolism, which is referred to as CKD-MBD (CKD-mineral and bone disorder). Homeostasis of bone mineral metabolism collapses with the decline of renal function, but various compensation mechanisms maintain blood Ca and P levels for protection against systemic organ damage in early-stage CKD. When the estimated glomerular filtration rate (eGFR) declines to about 60 mL/min/1.73 m^2^, the active vitamin D (1,25(OH)_2_D_3_) level starts to decline and parathyroid hormone (PTH) subsequently elevates. However, the fibroblast growth factor 23 (FGF23) level elevates earlier than these hormonal changes. Since the expression of Klotho, a coreceptor of FGF23, declines in the kidney in very early-stage CKD, FGF23 rises due to resistance to FGF23 signaling in the kidney [1]. FGF23 can increase the urinary phosphorus excretion rate (FEP = (urinary phosphorus/blood phosphorus)/(urinary creatinine/blood creatinine) × 100 (%)) as a phosphaturic factor and suppress activation of vitamin D, resulting in the major trigger in the course of CKD-MBD (Figure 1) [2].

Calciprotein particles (CPPs) are a complex of Ca, P, and proteins such as fetuin-A that have the major physiological function of transport of hydroxyapatite to the bone without crystallization in other tissues. The blood level of CPPs elevates with excess P and Ca, and this contributes to arteriosclerosis [3] and inflammatory response [4] in CKD. The CPP level increases in the G1 and G2 stages of CKD, just before the rise of FGF23 [3]. These clinical findings lead to the hypothesis that CPPs might induce FGF23, and this is supported by basic studies, as described below. We recently also confirmed this hypothesis in vitro and in vivo [5]. In addition, the CPP level correlates significantly with the levels of P and FGF23, but not Ca, and thus can rise even in a state of hyperphosphatemia and hypocalcemia associated with progression of CKD [6]. However, a further study is necessary to establish the mechanism of CPP formation in vivo. Sequential changes in CKD-MBD might involve CPPs and FGF23 for homeostasis of bone and mineral metabolism.

## 2. Physiology and Pathology of CPPs

### 2.1. Components of CPPs

CPPs consisting of mineral and fetuin-A and matrix GLA protein (Mgp) at 18%, 80%, and 2%, respectively, were first discovered in the blood of rats treated with alendronate [7]. Since Mgp is a protein with autocrine and paracrine effects, Ca, P, and fetuin-A are thought to be the major components of CPPs. Physiologically, CPPs transport hydroxyapatite material to the teeth and bones for growth and remodeling, without precipitating this material in soft tissues [8]. Fetuin-A, which is mainly produced in hepatocytes, is a glycoprotein of about 60 kDa that is abundant in extracellular fluid and has a concentration in the blood of 0.5–1.0 g/L in healthy people. Inactivation of the insulin receptor tyrosine kinase and transforming growth factor *β* pathways are well-known biological effects of fetuin-A, and severe ectopic calcification was found in fetuin-A-deficient mice [9, 10].

In vitro analysis showed that initial (primary) CPPs with diameter 60–75 nm are complexes of fetuin-A and a Posner cluster of Ca_9_(PO_4_)_6_, which is the minimum unit of calcium phosphate (diameter 9.5 Å) that exists as a colloid. Developed (secondary) CPPs include hydroxyapatite resulting from calcium phosphate crystallization over time in the core and are about 120–150 nm in size [11, 12]. The strong negative charges in the *β* sheet structure of the cystatin-like D1 domain of fetuin-A are thought to promote binding with calcium phosphate [11].

### 2.2. Metabolism of CPPs

The half-lives of primary and secondary CPPs are 149 and 45 min, respectively, in mice intravenously injected with fluorescently labeled synthetic CPPs. The particles are phagocytosed by F4/80- or CD68-positive macrophages and Kupffer cells in the liver and by macrophage receptor with collagenous structure- (MARCO-) positive macrophages in the spleen. Primary CPPs are also deposited in the liver, kidney, and bone marrow, and so it is assumed that CPPs are metabolized in these organs [13]. CPPs might be metabolized for physiological protection against organ damage resulting from their accumulation. Similar organ damage such as cardiovascular disorders occurs with excess production of CPPs in CKD-MBD and with the loss of fetuin-A, as described below.

### 2.3. Involvement of CPPs in Inflammatory Response and Cardiovascular Disorders

Only secondary CPPs induced cell necrosis, mineralization, and TNF-*α* mRNA and protein in experiments in primary human aortic vascular smooth muscle cells (VSMCs) treated with primary or secondary CPPs. These effects were exacerbated and attenuated by the administration of TNF-*α* and its inhibitor, respectively. These findings suggest that secondary CPPs might locally induce cytokines such as TNF-*α*, which may result in vascular damage [14]. Treatment with an inhibitor of CPP formation ameliorated vascular damage in rats fed an adenine-containing diet and reduced elevation of the blood CPP level and vascular calcification [15]. On the other hand, secondary CPPs were found in vascular lesions, in which CD68-positive macrophages phagocytosed secondary CPPs through scavenger receptor-A (SR-A) I/II in mice deficient in ApoE and fetuin-A [13]. Therefore, CPPs might be contributing to vascular calcification in these pathological animal models.

### 2.4. CPPs Induce FGF23 in Bone

FGF23 is a hormone that promotes P excretion in urine by suppressing type II sodium-dependent phosphate cotransporters (Npt2a and Npt2c), which are expressed on the luminal side of proximal renal tubular cells in the kidney. The blood FGF23 level elevates according to the balance of P for its homeostasis. In rat osteoblast-like cells (UMR-106) and mouse osteocyte-like cells (IDG-SW3), P in the medium upregulates FGF23 mRNA in a dose-dependent manner [16, 17]. However, a low Ca level suppresses this upregulation [5]. This finding was also confirmed in an animal model treated with a high phosphate diet [18]. Moreover, experiments in which blood Ca and P levels were controlled in mice lacking one or both genes for the Ca-sensing receptor and PTH revealed that the product of the Ca and P levels is the strongest factor regulating the blood FGF23 level [19].

FGF23 is composed of a signal sequence (24 amino acids), N-terminal core homology domain (155 amino acids), and a C-terminal domain (72 amino acids). It is inactivated by cleavage at the sequence of 176RXXR179, which is recognized by a proteolytic enzyme. Intact FGF23 and C-terminal FGF23 assays that measure only full-length and both full-length and C-terminal fragments, respectively, are available. Many clinical studies of CKD have shown a significant correlation of these assays with each other and with the elevation of P in the blood and progression of CKD, indicating the similar clinical significance of the two assays [20]. The blood CPP level is also significantly correlated with intact FGF23 and P levels in dialysis and nondialysis patients with CKD [6].

These basic and clinical findings suggest that CPPs (the complex of Ca and P), but not P itself, might be a physiological and pathological contributor to P-induced FGF23. However, it will be necessary to evaluate the in vivo effects of inhibitors of CPP formation on P-induced FGF23 to confirm this hypothesis. TNF-*α* and IL-1*β* elevated FGF23 mRNA and protein levels in IDG-SW3 cells [21], and similar findings were obtained following the administration of secondary CPPs [22]. Therefore, CPPs may induce inflammatory responses that both result in organ damage, including cardiovascular disease, and induce FGF23 in bone cells.

## 3. Clinical Significance of CPPs

The blood CPP level, the high sensitivity C-reactive protein (CRP) level, the coronary artery calcification score, and the aortic pulse wave velocity (aPWV) are all elevated with loss of renal function and are all correlated with each other [3, 4], while the blood fetuin-A level is negatively correlated with blood CRP, all-cause mortality, and cardiovascular death in patients with hemodialysis [23]. Therefore, consumptive loss of fetuin-A caused by excess CPP formation and inhibition of production of fetuin-A in the liver by uremic toxins such as indoxyl sulfate might also contribute to the high mortality in these patients [24].

A significant relationship between the blood CPP level and mortality has recently been shown in patients with CKD, and new CPP assays have been developed. The turbidity method using a nephelometer system has been applied for the evaluation of CPP status in serum, based on the measurement of the half time for maximal transition (T50) from primary to secondary CPPs in serum after adding Ca and P solutions. The T50 was significantly shorter in patients with hemodialysis than in healthy volunteers, and the T50 in fetuin-A-deficient mice was also shorter than that in wild-type controls [12]. A recent study also suggested that secondary CPPs are easily formed from precursors such as primary CPPs in CKD and that this might contribute to tissue and organ damage under stress in CKD [6]. T50 shortening is related to the high risk of death in patients in CKD stages G3 and G4 and in kidney transplant recipients [25, 26] and to all-cause death, myocardial infarction, hospitalization due to unstable angina pectoris, heart failure, and peripheral vascular disease in patients on hemodialysis [27].

**Figure 2:**
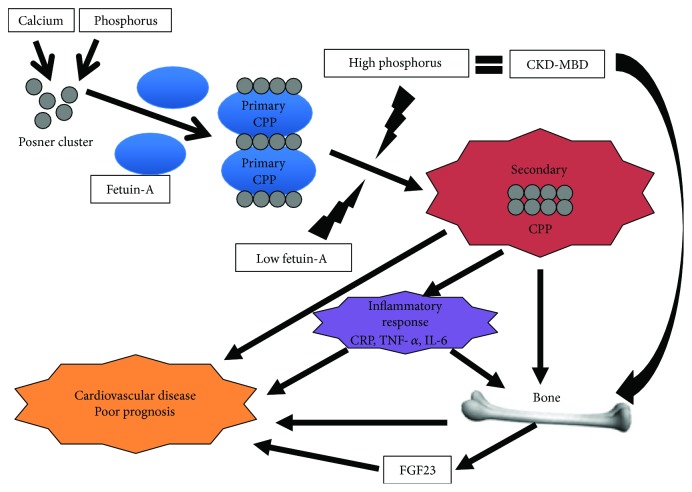
The physiological role of primary CPPs is accurate transportation of materials for the bones and teeth. Developed (secondary) CPPs may induce an inflammatory response through induction of TNF-*α*, IL-6, and CRP in CKD and may upregulate FGF23 in bone cells. These sequential abnormalities can contribute to the risk of cardiovascular disease and the poor prognosis in patients with CKD.

Methods for measuring primary and secondary CPPs in the blood by flow cytometry using fluorescently labeled probes have also been developed [28]. The secondary CPP level in the blood is significantly higher in hemodialysis patients than in healthy subjects, and P, FGF23, IL-1*β*, IL-6, and diabetes mellitus are significantly correlated with this level. These results suggest that secondary CPPs are a new biomarker for the pathological condition of CKD-MBD (Figure 2).

## 4. CKD-MBD Normalization by Surgery May Improve Clinical Outcomes (Table 1)

The major surgical treatments for CKD-MBD are kidney transplantation and parathyroidectomy. Both exert powerful effects that are not obtained in conventional dialysis or drug treatment, resulting in significant improvement of CKD-MBD. Since the CPP level is significantly correlated with the P level in patients with CKD [6], CPP formation may be suppressed following parathyroidectomy and kidney transplantation, which might markedly decrease the P level. However, further studies on the actual change in the CPP level after surgery are required.

The P level markedly decreases because the recovery of kidney function activates phosphaturic function following kidney transplantation in patients with end-stage renal failure. Most recipients can quickly withdraw from dialysis therapy, except in a case of delayed graft function. The Ca level also decreases immediately after kidney transplantation and reaches a normal level after about six months. Along with the recovery of active vitamin D synthesis in the grafted kidney, the Ca status is improved. However, high levels of PTH and FGF23 often persist, as described below [29], and a high PTH level in patients with advanced hyperplasia of the parathyroid gland, such as nodular hyperplasia, is present even years after renal transplantation [30].

The extremely high FGF23 level in patients with CKD-MBD is associated with all-cause mortality, risk of cardiovascular events, vascular calcification, cardiac hypertrophy, anemia, malnutrition, and chronic inflammation [31]. The FGF23 level starts to decrease about one week after transplantation but may not reach the normal range for almost one year [32]. However, renal transplantation improves most of the complications associated with the high level of FGF23 [33–37].

The rapid decrease in excess PTH after parathyroidectomy causes hypocalcemia and hypophosphatemia because of the rapid flow of Ca and P into the bone due to marked suppression of high turnover bone (hungry bone syndrome) [38]. Excess FGF23 also decreases [39], and these biochemical changes indicate a decreased CPP level after parathyroidectomy.

Many reports have shown that parathyroidectomy improves clinical outcomes such as all-cause mortality, cardiovascular events, vascular calcification, cardiac hypertrophy, anemia, malnutrition, and chronic inflammation [40–46]. Improved outcomes by kidney transplantation and parathyroidectomy are related to the improvement of CKD-MBD and particularly normalization of excess P, FGF23, and PTH. These effects may all contribute to the suppression of the secondary CPP formation, as a protective mechanism for inhibiting aggressive inflammatory activity [16].

## 5. Conclusion

CPPs have attracted recent attention in clinical and basic research as a new molecular marker involved in the pathology of CKD-MBD. Excess P intake from original phosphorus-rich food and from fast food, soft drinks, and food preservatives might enhance CPP formation and result in a poor prognosis for patients with CKD. The new findings on CPPs have improved the understanding of the pathophysiology of CKD-MBD and assist in the management of CKD-MBD for the prevention of complications such as cardiovascular disease. Surgical interventions may be most effective for improving clinical outcomes through amelioration of CKD-MBD, including reduction of the levels of FGF23 and CPPs.

## Figures and Tables

**Table 1:** The effects of surgical treatments on the major complications of CKD-MBD.

	Relations of biochemical parameters			Effects of surgical treatments	
	Calcium	Phosphorus	FGF23	Renal transplantation	Parathyroidectomy
All-cause mortality	Positive [47]	Positive [47]	Positive [31]	Improved [33]	Improved [40]
Cardiovascular events	Positive [48]	Positive [48]	Positive [31]	Improved [34]	Improved [41]
Vascular calcification	Positive [49]	Positive [49]	Positive [31]		Improved [42]
Cardiac hypertrophy	Positive [50]	Positive [51]	Positive [31]	Improved [35]	Improved [43]
Anemia	Positive [52]	Positive [52]	Positive [53]		Improved [44]
Malnutrition			Positive [31]	Improved [36]	Improved [45]
Chronic inflammation	Positive [54]	Positive [54]	Positive [54]	Improved [37]	Improved [46]
